# The Relationship between Resting Metabolic Rate and Body Composition in People Living with Overweight and Obesity

**DOI:** 10.3390/jcm13195862

**Published:** 2024-10-01

**Authors:** Evdoxia Gitsi, Alexander Kokkinos, Sofia K. Konstantinidou, Sarantis Livadas, Georgia Argyrakopoulou

**Affiliations:** 1 Diabetes and Obesity Unit, Athens Medical Center, 15125 Athens, Greece; sissykonstantinidou@gmail.com (S.K.K.); gargyrakopoulou@gmail.com (G.A.); 2 First Department of Propaedeutic Internal Medicine and Diabetes Center, Medical School, National and Kapodistrian University of Athens, Laiko General Hospital, 11527 Athens, Greece; akokkinos@med.uoa.gr; 3 Endocrine Unit, Athens Medical Center, 15125 Athens, Greece; sarantislivadas@gmail.com

**Keywords:** resting metabolic rate, body composition, liver steatosis, magnetic resonance imaging, obesity

## Abstract

**Background/Objectives:** Resting metabolic rate (RMR) is an important contributor of energy balance and displays a well-documented relationship with sex, age, race and fat-free mass (FFM) in the existing scientific literature. However, the impact of other body composition components such as fat and liver fat on RMR remains unclear. This study aims to investigate the correlation of RMR with body composition parameters in a sample of patients with overweight and obesity. **Methods:** Retrospective data of patients with overweight or obesity referred for magnetic resonance imaging of liver fat during the period 2018–2023 were utilized for this study. Demographic and anthropometric data were collected, including body composition parameters (body fat, muscle mass) and RMR measured by bioelectrical impedance and indirect calorimetry, respectively. **Results:** The final sample included 53 patients (66% male), with a mean age of 48 years (±11.2) and a mean body mass index (ΒΜΙ) of 38.5 kg/m^2^ (32.7, 44.7). Simple correlation models revealed that RMR was separately correlated with gender, age, BMI, muscle mass, and liver fat (all *p* < 0.05) but not with fat mass. When multiple regression models were employed, only muscle mass retained its statistically significant influence on RMR, while total and hepatic fat did not significantly affect RMR after controlling for other parameters (gender, age, muscle mass). **Conclusions:** These findings confirm the known correlation between muscle mass and RMR while highlighting the lack of association between total and hepatic fat and RMR in individuals with overweight and obesity.

## 1. Introduction

### 1.1. Overview of Overweight and Obesity Epidemic

In today’s global landscape, the undeniable surge of overweight and obesity to epidemic proportions, as highlighted by the World Health Organization, marks a significant public health crisis [1,2]. Obesity is a chronic complex disease characterized by excessive fat accumulation that can impair health, diagnosed by a body mass index (BMI) over/equal to 30 kg/m^2^, while overweight represents a condition of excess body weight typically defined by a BMI ranging from 25 to 29.9 kg/m^2^ [1]. According to recent worldwide trends in abnormal weight (underweight and obesity) published in The Lancet, in 2022, obesity was more prevalent than underweight in most countries for both women (89%) and men (73%), with a high level of statistical significance (posterior probability of at least 0.80) [3]. In Greece, findings from the EMENO National Health Examination Survey indicate that 37.6% of the adult population is living with overweight and 32.1% is living with obesity. When combined, these statistics reveal that approximately 7 out of 10 individuals live with excess body weight [4]. Consequently, this situation can be characterized as an epidemic, given its association with a range of chronic diseases, including diabetes, hypertension, cardiovascular disease, and cancer [5]. Moreover, obesity adversely affects mental well-being, increasing the risk for depression, anxiety, and low self-esteem [6]. Social stigma and discrimination further compound the challenges faced by individuals living with obesity, underscoring the need for holistic approaches to address both the physiological and psychosocial aspects of this condition [7].

Understanding the causes of overweight and obesity is crucial for effective management. The etiology of overweight and obesity is multifaceted, encompassing a diverse spectrum of factors, including lifestyle (mostly diet and physical activity), social, and environmental aspects (obesogenic environment, country, religion, socioeconomic status, and education), as well as biological factors (age, sex, genotype, epigenetic modifications, and microbiota composition), among others [8]. Regardless of the complexity of factors contributing to obesity, its pathogenesis entails two interconnected yet separate mechanisms [9]. The first process involves a chronic dysregulation of energy balance, which involves excessive calorie consumption (food intake) and/or insufficient or inadequate calorie expenditure (metabolic and physical activity) [10]. The second process is about the resetting of the body weight “set point” or, more accurately stated, the “set range” at an elevated level [9]. This phenomenon elucidates why weight lost through dietary and lifestyle changes tends to be regained over time, posing a substantial obstacle to successful obesity management [9].

### 1.2. Understanding RMR and Its Measurement Techniques

As previously mentioned, obesity results from chronic imbalanced energy levels, wherein energy intake surpasses energy expenditure [11]. Thus, in order to manage obesity and facilitate weight loss, a switch to achieving a negative energy balance is fundamental [12]. Energy balance is determined by the disparity between energy intake from food and total energy expenditure (TEE) [12]. Total energy expenditure comprises three main components: resting metabolic rate (RMR), diet-induced thermogenesis, and physical activity or activity thermogenesis [13].

RMR stands as the primary contributor to daily energy expenditure for most individuals, accounting for 60–80% of its total value and varying based on the individual’s activity level [13,14]. RMR, often referred to as resting energy expenditure (REE), is typically defined as the energy expended by the body in a resting state [15]. This encompasses the energy expenditure while the individual is awake, in a post-absorptive and thermoneutral condition and has refrained from exercise for approximately 12 h [15,16,17]. Diet-induced thermogenesis, influenced by meal composition, typically accounts for 10–15% of TEE, with protein causing the highest thermogenic effect, while activity energy expenditure varies widely, from less than half of basal energy in sedentary individuals to more than double in athletes [18,19,20].

RMR is the primary determinant of TEE and therefore, plays an important role in shaping the overall energy balance of non-athletic individuals, including those with overweight and obesity. Accurate measurement of RMR is essential in clinical practice for better clinical decision-making and the selection of optimal therapeutic approaches. However, this process, which is performed via direct or indirect calorimetry, often requires skilled technicians and sophisticated methodologies, which can be costly and difficult to carry out [21]. Due to these challenges, RMR measurement is often impractical in many clinical and community settings [21]. RMR prediction equations utilize easily obtainable variables such as age, height, and body weight. Unfortunately, these equations only explain between 50% to 75% of the variability in RMR [22]. There is ongoing debate regarding the validity of these equations, as they may not accurately estimate caloric needs, given their application in significantly different populations and conditions than they were originally intended for [23,24,25]. Additional factors such as body composition, ethnicity, medications, and external temperature have been suggested to play a role in determining RMR [26,27]. Furthermore, RMR prediction equations tend to misclassify certain populations, including children and adults with obesity, critically ill patients, and individuals with eating disorders [25]. An additional factor that influences RMR is the implementation of energy restriction, as observed in weight loss interventions through reduced energy intake. This practice, named metabolic adaptation, leads to a decrease in RMR as a biological response to energy restriction and may elucidate the difficulty in properly calculating patient’s energy needs, as well as maintaining weight loss associated with low-calorie diets [28]. Given the absence of consensus on the most suitable equation for accurately assessing caloric requirements in people with obesity, both before and after weight loss interventions, and the substantial divergence among equations, it is advisable to measure RMR rather than relying on estimation [29].

Indirect calorimetry stands as the gold standard for assessing RMR in clinical settings [30]. This method relies on measuring the intake of oxygen and the release of carbon dioxide, which are then used to compute the respiratory exchange ratio (RER), calculated as the ratio of carbon dioxide production (VCO_2_) to oxygen consumption (VO_2_). The RER is then utilized to estimate substrate oxidation by comparing the calculated RER values with the recognized RERs associated with the complete oxidation of macronutrients. An RER value around 0.7 indicates fat as the primary fuel source, while a value of 1.0 suggests carbohydrate utilization, and values between 0.7 and 1.0 indicate a mix of both fat and carbohydrate as the predominant fuel sources. A mixed diet corresponds to an RER of approximately 0.8–0.85 [31]. Calories expended by a person in a seated position are determined by the principle that each liter of oxygen consumed equates to 3.9 kilocalories expended, while each liter of carbon dioxide produced accounts for 1.1 kilocalories expended. When an awake and alert person is at rest in the postabsorptive state, the energy expenditure measured by indirect calorimetry closely represents REE or RMR. RMR is often used equivalently with BMR, which refers to the minimal heat production observed 12 to 18 h after food ingestion while at complete rest. Valid measurement of BMR occurs during sleep, in a thermoneutral environment, with the individual lying down. RMR encompasses BMR plus an increase due to awakening and the thermic effect of food, making it approximately 10% higher than BMR [30].

Conducting indirect calorimetry offers a crucial advantage by preventing overfeeding in patients, particularly those with overweight and obesity, through precise assessment and management of their energy needs [32]. Portable devices designed to evaluate RMR have been created for practical use in office and clinic environments and have undergone validation in adult populations [28]. While a conventional indirect calorimeter assesses an individual’s expired gas volume and the proportions of CO_2_ and O_2_, thus estimating RMR, smaller portable versions lack a CO_2_ sensor. Instead, they measure O_2_ consumption, operating on the assumption that CO_2_ production approximately equals 85% of O_2_ consumption [28].

### 1.3. Factors Correlated with RMR and Their Significance

**Figure 1:**
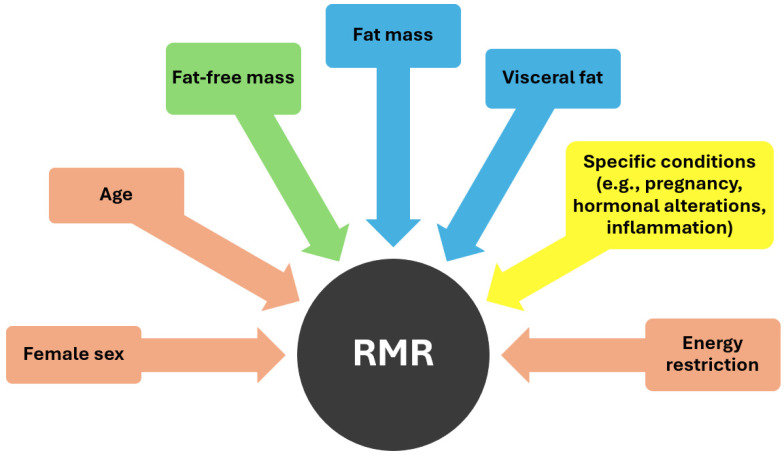
Factors influencing RMR (pink color: negative effect, green color: positive effect, blue color: uncertain effect, yellow color: dependent on specific conditions).

As demonstrated in Figure 1, various factors influence RMR, including age and sex. Basal metabolism has been observed to decrease by 1–2% per decade from the age of 20 to 75 years, with this aging process often accompanied by the substitution of muscle mass with an increase in fat mass (FM) [26,33]. Moreover, RMR tends to be higher in men compared to women probably because of the higher proportion of fat-free mass (FFM) in the former [15]. FFM serves as the primary determinant of resting energy expenditure [34]. Metabolically, FFM comprises two distinct components: metabolically active FFM and low-metabolic-rate tissues or organs. Metabolically active FFM includes both muscle and non-muscle organ mass, with skeletal muscle and certain organs such as the brain and visceral organs differing significantly in their masses, growth rates, and rates of energy expenditure [35]. High-metabolic-rate organs such as the brain, heart, liver, and kidneys exhibit metabolic rates per kilogram of organ weight approximately 10–20 times higher than the body as a whole, while skeletal muscle, considered a low-metabolic-rate tissue, has significantly lower metabolic rates per kilogram [34]. Consequently, the majority (approximately 70–80%) of REE in adults is attributed to organs despite comprising only a small percentage of total body weight. Some other specific factors that can affect metabolism are pregnancy, which necessitates increased energy expenditure to support fetal growth and maternal tissue expansion, as well as various hormones like thyroxine, catecholamines, and insulin which may also contribute to an increased energy expenditure [29].

An additional factor that influences RMR is the implementation of energy restriction, as observed in weight loss interventions through reduced energy intake. This practice leads to a decrease in RMR and may elucidate the difficulty in maintaining weight loss associated with low-calorie diets, reflecting the biological response to energy restriction, otherwise known as metabolic adaptation [36]. In the realm of weight loss interventions, metabolic adaptation emerges as a formidable obstacle to success. More accurately, metabolic adaptation refers to physiological decreases in energy expenditure and RMR subsequent to weight loss, surpassing the expected changes solely attributable to alterations in body composition [31]. It often accounts for the variance between observed and predicted body weight loss and can be quantified by the disparity between measured RMR and predicted RMR post-weight loss [19]. The progression of metabolic adaptation during weight loss may be also predicted by the extent of fluctuations in hormone secretion levels, including leptin, ghrelin, glucagon-like peptide-1 (GLP-1), and cholecystokinin (CCK) [28]. These hormonal changes often lessen the sensation of satiety, potentially fueling increased food consumption. Consequently, this phenomenon significantly contributes to the prevalent issue of weight regain following initial loss in individuals living with obesity [28].

While FFM is strongly positively correlated with RMR according to current literature, FM could also potentially influence RMR, especially in populations with excess weight who, by definition, have higher adiposity levels [19]. Elevated inflammation and cytokine release, coupled with heightened reactive oxygen species (ROS) generation associated with high levels of adiposity, likely contribute to the elevation in BMR. This, in turn, leads to disrupted mitochondrial function and finally in surplus heat production and a higher level of metabolic activity [29]. Some studies suggest that the visceral fat component may play a more significant role than muscle mass in the context of excess weight [37]. This can be explained by the increased metabolic activity that visceral fat exhibits due to increased sympathetic activity and blood flow, as well as to higher lipolysis rate, along with the presence of chronic, low-grade inflammation commonly observed in individuals with central obesity and metabolic syndrome [29,37].

As noted above, despite being less metabolically active than FFM, FM appears to be a separate determinant of REE, particularly in individuals with substantial amounts of adipose tissue [38]. Considering the higher REE in individuals with obesity, the disparity in their FM compartment should be taken into account when formulating clinical weight management plans [27]. To further enhance comprehension, let us consider the case of a 30-year-old female with a BMI of 59.6 kg/m^2^. Her measured REE stands at 2726 kcal/day, whereas the Harris–Benedict equation predicts her REE to be 2274 kcal/day (16.6% below the measured value), and the lean mass-based Katch–McArdle equation estimates it to be 1752 kcal/day (35.7% below the measured value). These predictive equations may potentially underestimate REE due to the omission of FM characterization, a factor that holds particular relevance for individuals with elevated BMIs [39].

Due to the aforementioned limitation of existing predictive equations, some studies suggest that roughly 10% or more of a woman’s estimated REE may remain unaccounted for due to the metabolic rate of FM [40]. The concept that FM can substantially contribute to REE, especially in individuals with high adiposity is likely to be amplified in women with greater body fat percentages. Recognizing the contribution of FM to REE in populations with overweight/obesity, averaging around 10%, could allow for adjustments to predictive equations [40]. For instance, by increasing the Harris–Benedict predicted value by 10% to 2501 kcal/day (8% below the measured value), more accurate calorie prescriptions may be achieved. This adjustment can aid healthcare practitioners in ensuring adequate caloric prescriptions, thereby helping to mitigate undesirable reductions in REE, such as adaptive thermogenesis [39]. However, stronger correlation data are needed to enhance our understanding of RMR parameters, which will enable us to refine the individualized therapeutic strategies used for effective obesity management. This study aims to examine potential correlations between body composition factors, including muscle mass, fat mass and liver fat, and RMR in individuals with overweight or obesity.

## 2. Materials and Methods

### 2.1. Patients (Inclusion and Exclusion Criteria)

Patient data from the Diabetes and Obesity unit of Athens Medical Center, spanning from 2018 to 2023, were retrospectively reviewed. The search targeted individuals with overweight or obesity, regardless of their diabetes mellitus status, as determined by their BIA measurements during their initial visit, prior to any structured weight loss intervention. These patients had also undergone RMR testing using indirect calorimetry during their visit, which is standard practice in the obesity department as part of their nutritional assessment, and had received liver fat quantification through magnetic resonance imaging (MRI). The focus was on patients who presented in the morning for resting metabolic rate measurement and were referred for MRI examination due to clinical suspicion of elevated visceral fat. Before their visit, patients were informed of the key prerequisites for measurements, including a complete 12 h fast from any type of food and drink (including water) and refraining from tobacco use. Moreover, they were asked to abstain from strenuous physical activity for 12 h.

**Figure 2:**
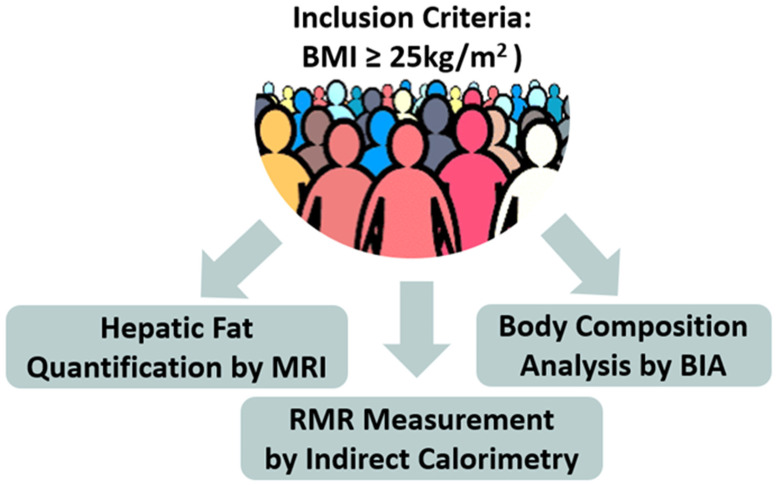
Schematic representation of the study methodology (BIA = Bioelectrical Impedance Analysis, MRI = Magnetic Resonance Imaging, RMR = Resting Metabolic Rate).

The participant selection criteria, comprising both inclusion and exclusion criteria, were as follows:

Inclusion Criteria (Figure 2)

- Provision of patient’s consent after being informed prior to any data collection and analysis.
- Diagnosis of overweight or obesity, as determined by calculated BMI (criteria for overweight: 25–30 kg/m^2^, criteria for obesity: ≥30 kg/m^2^).
- Performance of all the examinations described in the study protocol (measurement of body composition by BIA, measurement of RMR via indirect calorimetry, measurement of liver fat by MRI) during the initial visit, prior to any weight loss intervention.
- Strict adherence to the requirements for the aforementioned measurements.
- Exclusion Criteria
- Any disorder that, in the investigator’s judgment, may jeopardize compliance with the measurements needed for the study.

### 2.2. Description of the Evaluation Performed

Upon arrival in the clinic for the initial visit, as shown schematically in Figure 2, participants underwent:

- Anthropometric measurements: height (cm) and weight (kg).
- Body composition analysis via bioelectrical impedance (BIA) using the TanitaTBF-300A analyzer, (Tanita Corporation, Tokyo, Japan) [41]. The following parameters were obtained from BIA: weight [kg], BMI (calculated as the ratio of body weight [kg] to the square of height [m^2^]), percentage of body fat [%], total body water (TBW) [%], body fat [kg], muscle tissue mass [kg], visceral fat level [kg], and BMR [kcal].
- RMR measurement via indirect calorimetry: The Cosmed FitMate system device, version 2.3, a validated and reliable tool for measuring oxygen consumption and RMR through indirect calorimetry in adults was utilized in this study [42]. The assessment occurred while participants were lying down in a well-ventilated and low-lit room with moderate temperature conditions. This examination was conducted in the morning following 7–9 h of sleep. Before the assessment, participants were given a few minutes to relax in a seated position to ensure optimal measurement conditions. Subsequently, they were instructed to wear a face mask provided by the FitMate system. The assessment lasted approximately 15 min, with the first 3 min discarded, and was carried out in a controlled environment to minimize external disturbances. Throughout the assessment process, an investigator or expert healthcare professional closely monitored the progress on the FitMate screen.
- Liver fat quantification via MRI: hepatic steatosis was assessed using a standardized MRI protocol, which included axial T1-weighted imaging from the abdominal–pelvic regions and axial proton density fat fraction (PDFF) imaging through the liver. MRI is recognized as the gold standard imaging technique for both qualitative and quantitative evaluation of hepatic steatosis [42]. The sensitivity and specificity of MRI for detecting histologically confirmed steatosis (≥5%) are well documented, ranging from 76.7% to 90.0% and 87.1% to 91%, respectively [43,44]. MRI measurements of liver fat provide valuable insight into abdominal or visceral fat, which includes adipose tissue located around vital organs such as the liver, stomach, and intestines. This type of fat correlates strongly with central obesity and metabolic complications, making it a critical factor in the study of obesity-related conditions [43].

Each participant provided voluntary informed consent prior to any study-related analysis of their data and received a unique numerical code to ensure anonymity. Personal data were kept confidential and only accessible to the research team.

### 2.3. Study Design

**Figure 3:**
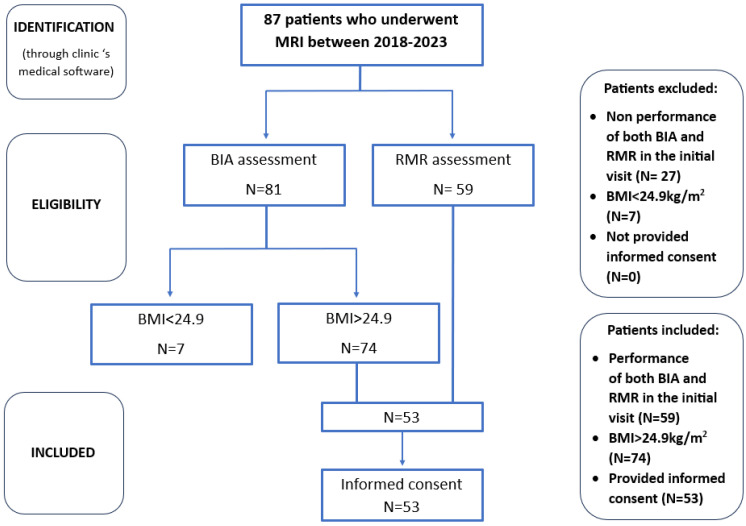
Box diagram for patient selection process.

After a comprehensive evaluation of the patient database, data were collected for a sample of 53 individuals who met the specified criteria. This sample size was deemed sufficient given the limited scope of the study population, particularly considering that liver fat quantification is not a routine procedure and its prescription must prioritize cost-effectiveness. Furthermore, as a pilot observational study intended to provide preliminary insights for future investigation, this sample size is considered adequate for drawing initial conclusions to address our research questions. The patient selection process is demonstrated in Figure 3.

The data collected were the following: unique participant IDs, gender, age, weight, height, BMI, FM, muscle mass, visceral fat, hepatic fat, predicted RMR using equations, actual RMR using indirect calorimetry. After data collection, statistical analysis was performed to examine correlations and address the research questions of the study.

### 2.4. Statistical Analysis

For describing the characteristics of the participants, statistical measures of central tendency and dispersion were used. Specifically, results were presented using the mean ± standard deviation for continuous variables and respective frequencies (%) for categorical variables. Normality of the distribution of continuous variables was assessed using the Shapiro–Wilk normality test. Subsequently, necessary correlation analyses were performed. The Pearson method (for parametric data) and the Spearman method (for non-parametric data) were utilized to estimate the correlation coefficient between the values (quantitative parameters) among different measurements. For constructing simple and multiple dependency models, depending on the distribution of quantitative parameters, appropriate analyses (linear, robust, and local polynomial regression) were used. Finally, a significance level of *p* < 0.05 was set as the statistical significance threshold. Statistical analyses were carried out with the SPSS statistical package version 28.0.1.1 and STATA version 12.0.

## 3. Results

### 3.1. Descriptive Statistics

**Figure 4:**
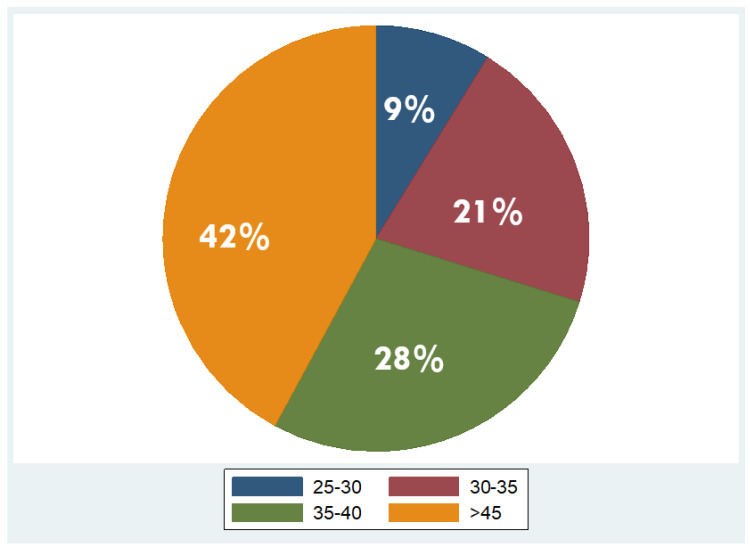
Distribution of overweight and obesity classes in the study population (overweight: BMI 25–29.9 kg/m^2^; obesity class I: BMI 30–34.9 kg/m^2^; obesity class II: BMI 35–39.9 kg/m^2^; obesity class III: BMI ≥ 40 kg/m^2^).

**Figure 5:**
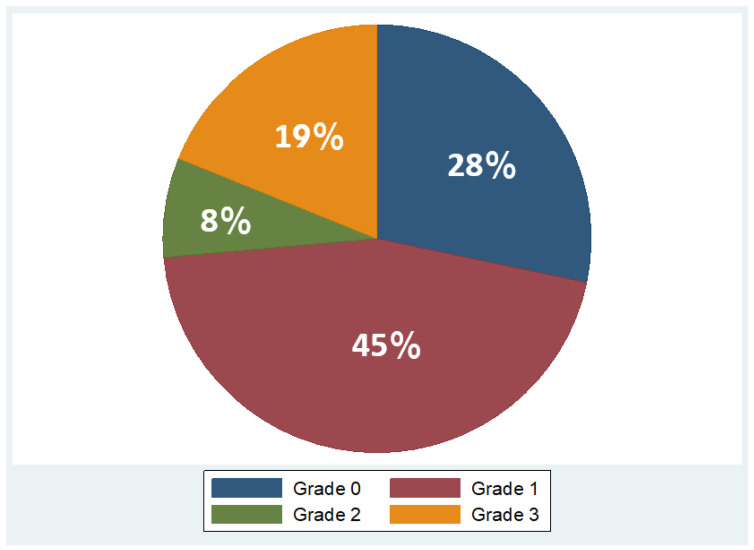
Variation in fat grades detected by MRI in study population (Grade 0: <6.3%, Grade 1: 6.3 –17.4%, Grade 2: 17.5 –22%, Grade 3: ≥22.1%).

Overall, 53 patients were included, of which 35 were males (66%) and 18 females (34%), with a mean age of 48 years ± 11.2 (mean ± standard deviation, SD). Regarding BMI, the median BMI stood at 39 kg/m^2^, with the 25th and 75th percentiles at 33 and 45 kg/m^2^, respectively. The percentages of the participants in different BMI categories were as follows: 9% in overweight (BMI 25–29.9 kg/m^2^), 21% in obesity class one (BMI 30–34.9 kg/m^2^), 28% in obesity class two (BMI 35–39.9 kg/m^2^), and 42% in obesity class three (BMI ≥ 40 kg/m^2^). As concerns liver fat distribution quantified by MRI, 28% of the participants were in grade 0 (<6.3%), 45% in grade 1 (6.3–17.4%), 8% in grade 2 (17.5–22%), and 19% in grade 3 (≥22.1%) [45]. Measured RMR with indirect calorimetry averaged at 2125 kcal (SD ± 501 kcal), whereas predicted RMR from equations used in BIA analysis averaged at 2210 kcal (SD ± 432 kcal). More descriptive statistics of the study population are shown in Table 1 and in the pie charts in Figure 4 and Figure 5.

### 3.2. Correlation Analysis

The Pearson correlation coefficients presented in Table 2 illustrate the associations between RMR and various factors including age, anthropometric data, and body composition.

The correlation between RMR and age was found to be moderately negative (r = −0.39, *p* = 0.004), indicating that as age increases, RMR tends to decrease. In contrast, RMR demonstrated a strong positive correlation with BMI (r = 0.43, *p* = 0.0008), muscle mass (r = 0.78, *p* < 0.001) and visceral fat (r = 0.61, *p* < 0.001), suggesting that higher BMI, muscle mass, and visceral fat are associated with higher RMR values. However, the correlation between RMR and FM percentage from BIA was very weak (r = −0.06, *p* = 0.69), indicating a negligible linear relationship between these variables. Additionally, the correlation between RMR and liver fat percentage from MRI was weak (r = 0.23, *p* = 0.09), suggesting a minimal association between RMR and liver fat percentage.

With regard to the correlation between actual (RMR measured by indirect calorimetry) and predicted RMR (RMR predicted by BIA equations), outcomes reveal a strong positive linear relationship (r = 0.78, *p* < 0.001), implying that there is no significant difference in the results of the BIA and indirect calorimetry measurements of RMR in the sample.

### 3.3. Linear Regression Analysis

For the evaluation of the potential correlation of RMR with body composition factors in the sample of individuals with overweight and obesity, a linear regression analysis was conducted. In this analysis, demographic factors (age, gender) were taken into account as possible confounding variables, as well as anthropometric characteristics (fat mass, muscle mass) and metabolic characteristics (liver fat).

**Figure 6:**
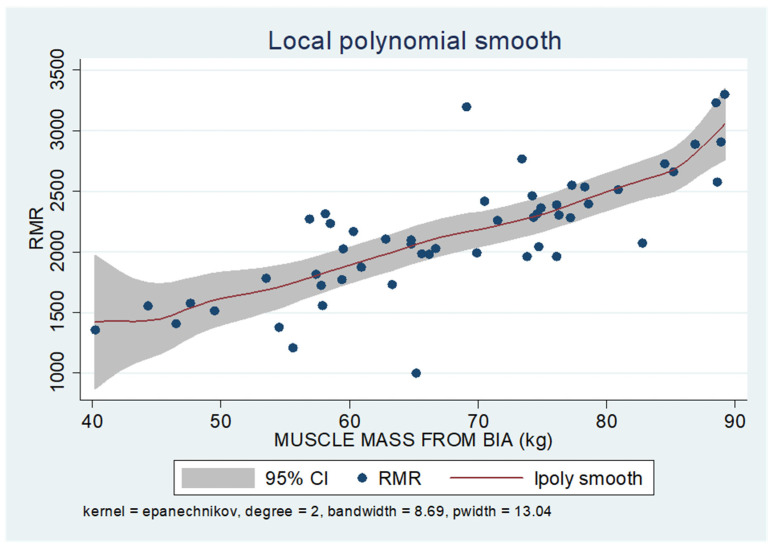
Local polynomial regression analysis: nonlinear association between muscle mass and RMR (*p* < 0.05).

**Figure 7:**
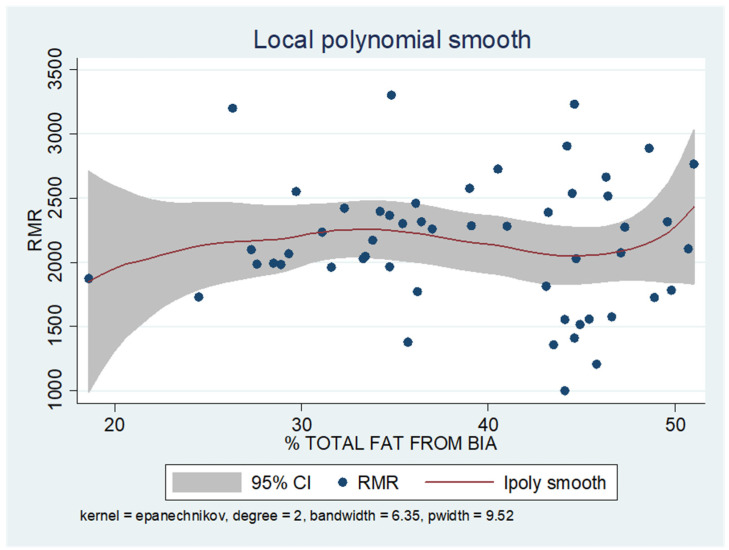
Local polynomial regression analysis: nonlinear association between total fat mass and RMR (*p* > 0.05).

**Figure 8:**
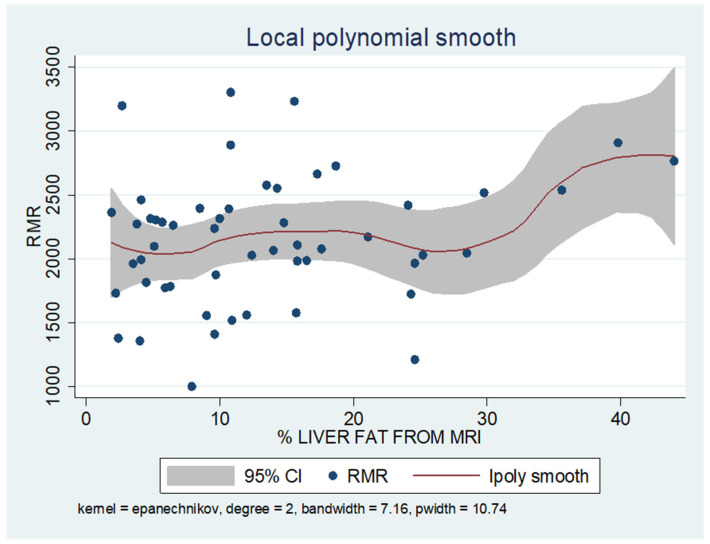
Local polynomial regression analysis: nonlinear association between liver fat and RMR (*p* > 0.05).

As shown in the unadjusted regression model in Table 3 (Model 1), gender appeared to be significantly correlated with RMR, with males in the sample exhibiting a 636 kcal higher RMR than females. After adding age to the model (Model 2), gender continued to retain its statistical significance in determining RMR, along with age, where an increase of 10 years resulted in a decrease in RMR by 130 kcal based on our findings. Subsequently, with the addition of body composition factors (fat mass, muscle mass, and liver fat), only muscle mass retained its statistical significance in determining RMR. Specifically, in the final linear regression model (Model 3), it appears that an increase in muscle mass by 1 kg leads to an increase in RMR by 24 kcal (*p* = 0.002). Regarding total fat, it was found that an increase in it results in a mild increase in RMR without statistical significance, while for liver fat, a neutral to slightly inverse but statistically insignificant relationship was observed, indicating that an increase in liver fat does not affect RMR or leads to a minimal decrease. Local polynomial analysis graphs revealing the associations between muscle mass, fat mass, and liver fat with RMR are presented in Figure 6, Figure 7 and Figure 8.

For the evaluation of the goodness of fit of the final model correlating RMR with body composition factors, the coefficient of determination (R^2^) was computed. The R^2^ value was 0.631, which implies that 63,1% of the variance in RMR can be explained by the variables included in the model, while the remaining percent may be attributed to unknown factors or inherent variability.

To uncover non-linear relationships, local polynomial regression analysis was employed. As depicted in the corresponding plots, muscle mass exhibits an almost perfectly linear and positive relationship with RMR, whereas the linear relationship seems to be absent in the plots of fat mass/RMR and liver fat/RMR. In the latter two relationships, it appears that there is a neutral to slightly positive relationship up to a certain threshold (~45% total fat and 30% liver fat), beyond which the trajectory concerning RMR increases more sharply.

## 4. Discussion

In the present study, the relationship between RMR and known body composition factors, particularly total fat mass, muscle mass, and liver fat, was examined in a sample of patients with overweight and obesity. This correlation was investigated both in terms of direction and magnitude of the effect of each factor on RMR while also considering gender and age as potential confounding variables that might mediate this relationship, thereby weakening the independent influence of body composition on RMR. The main finding of this study was the strong positive correlation between muscle mass and RMR, with no impact observed for total FM or liver fat on RMR at a statistically significant level in the sample of 53 patients analyzed, aligning with the existing scientific literature. The limited impact of both total FM and liver fat on RMR was evident not only in simple paired correlations but also in the ultimate model of multiple linear regression. With regard to the scientific literature, despite the well-documented relationship between FFM and REE, the impact of FM on REE remains uncertain, especially across different levels of adiposity.

A relative study conducted by Lührmann et al. delved into the impact of various body composition parameters on the RMR of elderly individuals aged over 60, encompassing a spectrum of BMI categories from normal to class II obesity [45]. Their findings revealed that a substantial portion of the RMR variability could be elucidated by FFM, exhibiting notable coefficients of determination, R^2^ of 0.54 for women and 0.44 for men. This strong correlation may be attributed to the significant size and metabolic activity of FFM [34]. In our investigation, we observed a higher R^2^ value of 0.61 solely attributable to muscle mass, underscoring its significant influence. However, this still suggests the existence of genetic factors beyond muscle mass that likely contribute to the remaining variability in RMR.

Moreover, Lührmann et al.’s study highlighted the contribution of FM, where, as a singular parameter, it elucidated an additional 3% and 2% of RMR variability in women and men, respectively [45]. However, in their stepwise multiple regression analysis, when incorporating both body composition and fat distribution, only FFM and waist-to-hip ratio emerged as significant predictors of RMR in both genders. Beyond FFM, waist-to-hip ratio accounted for 6% in women and 8% in men of the RMR variability. This finding emphasizes the potential influence of FM distribution on RMR, suggesting that elevated RMR could be associated with increased abdominal fat, due to its heightened metabolic activity.

A slight correlation between waist-to-hip ratio and RMR was also found in another study involving women with obesity in premenopause. At the same time no significant link was reported between RMR and patterns of visceral fat distribution, as assessed by computed tomography (CT) scans [37]. Authors suggested that rather than visceral fat, the overall body composition, encompassing both FFM and body fat, has a greater impact on RMR. Waist-to-hip ratio, which is largely influenced by genetics, might contribute to this body composition. A possible interpretation of this relationship is the presence of fast-twitch muscle fibers, commonly found in individuals with central obesity. These fibers utilize anaerobic metabolism, leading to the production of lactic acid and subsequent conversion into glucose in the liver, thus elevating RMR by increasing energy expenditure.

Hagedorn et al. conducted indirect calorimetry in females with higher levels of obesity to identify the primary factors influencing RMR [29]. They discovered that waist circumference (WC), mid-arm circumference (MAC), and mid-arm muscle circumference (MAMC) were significantly and positively linked to RMR. WC is a robust indicator of visceral fat, whereas the association of MAC and MAMC with RMR could be attributed to the increased fat accumulation in the mid-upper arm among individuals with obesity, which mirrors the existence of adipose tissue in other body regions.

In a retrospective study involving 114 subjects without diabetes and a BMI greater than 35, half of whom had metabolic dysfunction-associated steatotic liver disease (MASLD), researchers found a correlation between increased liver fat and higher RMR. Specifically, they observed that individuals with hepatic steatosis, as determined by CT, exhibited significantly higher mean RMR, of 300 kcal at baseline compared to those without steatosis [36].

However, our study diverges from previous findings regarding the minor positive impact of central fat distribution, and possibly total body fat, on RMR, as we did not observe a significant correlation between either FM or liver fat, which is a component of abdominal fat, and RMR.

Our findings from local polynomial smoothing suggest a potential correlation between FM and liver fat with RMR after reaching a specific threshold. However, the reliability of these findings is compromised by the limited number of observations exceeding these thresholds. It is reasonable that higher percentages of body fat lead to a decrease in skeletal lean mass contribution and an increase in FM contribution to adjusted REE. In other words, FM could account for a greater portion of the variation in REE among people with varying levels of adiposity. One study has attempted to explore the influence of adiposity (fat mass) on REE in women. The authors indicated a linear relationship between FM and REE until adiposity reaches 40–50% body fat, suggesting a potential reduction in the metabolic rate of adipose tissue with increasing adiposity [27].

Another intriguing research inquiry involves examining whether the regional distribution of FFM influences RMR. This was assessed by a recent comparative study including both Caucasian and African American populations [21]. Upon accounting for age and total body FM, it was revealed that FFM in the upper body (encompassing arms and trunk) positively correlated with measured RMR in Caucasians (*p* <  0.005), contrasting with the insignificant impact observed in African Americans. This disparity was attributed to smaller organ sizes in African Americans, leading to lower energy expenditure during rest. However, in African Americans, FFM in the lower body (specifically legs) exhibited a positive association with measured RMR (*p*  <  0.005). This discrepancy underscores the distinct metabolic profiles between ethnic groups, suggesting that non-Hispanic White women tend to possess larger metabolically active organs within the trunk region compared to African American women, potentially contributing to variations in resting energy expenditure between the two groups and even to varying challenges in maintaining a normal weight. It is noteworthy that the mean BMI of subjects in this study was <30 kg/m², which may limit its generalizability to populations with obesity, while further studies are needed to elucidate the potential interplay of racial factors and body composition in regulating metabolic rate. Furthermore, in the aforementioned study, researchers found that for each kilogram gain in FFM, RMR was augmented by 28 kcal/day (*p* < 0.0001) [21]. Our own research yielded similar results, showing an increase of 24 kcal/day.

It is noteworthy that in the final model, the significance of other demographic factors diminished, suggesting that muscle mass acts as a mediator in the relationship between age and RMR. Specifically, as individuals age, their muscle mass tends to decline, thereby impacting RMR. Furthermore, this mediation extends to gender disparities, as males naturally possess greater muscle mass compared to females. Previous research has indicated that basal metabolism tends to decrease by 1–2% per decade from ages 20 to 75, with aging being accompanied by a noticeable replacement of a portion of muscle mass with an increase in FM [22]. Nevertheless, studies suggest that the decline in REE associated with aging is not solely attributed to alterations in body composition, indicating the involvement of other metabolic changes [23]. Another possible explanation for the variance in our findings could be that the average age of our participants was slightly lower than that of subjects in earlier studies that reported an association between age and RMR [22].

Moreover, our findings revealed a strong positive linear relationship between actual-measured by indirect calorimetry and predicted by BIA equations RMR, which underscores the effectiveness and reliability of the equations used for prediction prior to any weight loss intervention. According to another study, adjustments for multiple comparisons showed that the Mifflin–St.Jeor equation either overestimated or underestimated RMR in Caucasians. Moreover, the Cunningham equation underestimated caloric needs for this group, while the Harris–Benedict equation significantly overestimated caloric expenditure [21]. Consistent with findings from other studies, the Mifflin–St.Jeor equation emerges as the preferable choice for estimating energy requirements in Caucasian patients with obesity when direct measurement of REE is not feasible [24,25]. A good estimate of actual RMR values holds significant implications for conducting clinical assessments, prescribing medical nutrition therapy, and managing weight, within the context of an individualized and more effective approach [30].

Despite the insights gained from our study, it is imperative to acknowledge both its strengths and limitations to ensure a comprehensive understanding of the findings. As regards possible limitations, the observational design of our study precludes definitive conclusions regarding causality. Furthermore, the relatively small sample size may have limited the generalizability of our findings. Additionally, we should consider the limitations of the device used for measuring body composition. BIA is susceptible to various factors, including body hydration level and geometry, potentially leading to inaccuracies in body composition estimation, particularly in populations with obesity [32]. Notably, in individuals with obesity, the higher hydration of FFM can result in an overestimation of FFM and underestimation of FM when using this method [32]. Additionally, the presence of substantial adipose tissue alters electrical resistance, necessitating the use of higher frequency currents than the standard 50 kHz [46]. The scientific literature suggests that the BIA method may be reliable in patients with a BMI below 34 kg/m^2^. At higher BMI values, obtaining consistent and accurate body composition analyses becomes challenging. Therefore, cautious interpretation is warranted when employing BIA to assess body composition in this population.

As regards the strengths, our study benefits from the utilization of the FitMate system, a validated and reliable method of indirect calorimetry for measuring oxygen consumption and RMR in adults [47]. Additionally, MRI was employed, which is widely regarded as the gold standard imaging modality for both qualitative and quantitative assessment of hepatic steatosis with high sensitivity and specificity for detecting histologically confirmed steatosis (≥5%) [42,43,47]. By integrating these robust measurements, our study offers accurate assessments of the metabolic and hepatic health of our study population.

## 5. Conclusions

In conclusion, the present study revealed a robust positive correlation between muscle mass and RMR, while no significant impact was observed for total fat mass or liver fat on RMR. Our findings underscore the significant role of muscle mass in determining RMR, highlighting the need for further investigation into how other factors, such as fat distribution, may influence metabolic rate. Understanding these dynamics has significant potential to enhance outcomes from tailored therapeutic approaches and help prevent weight regain. Accordingly, healthcare professionals can develop more effective interventions focused on optimizing metabolic health and supporting sustainable weight management, thereby addressing the growing obesity epidemic and improving overall quality of life.

## Figures and Tables

**Table 1:** Descriptive statistics of the study population.

Study Population Characteristics	Values
Age (years), mean ± SD	48 ± 11
Sex (% male)	66
BMI (kg/m^2^), median, 25th and 75th percentiles	39 (33, 45)
Fat mass (%, from BIA), median, 25th and 75th percentiles	39 (33, 45)
Fat mass (kg, from BIA), mean ± SD	47 ± 17
Muscle mass (kg, from BIA), mean ± SD	68 ± 12
Visceral fat (kg, from BIA), median, 25th and 75th percentiles	24 (19, 28)
Liver fat (% from MRI), (median, 25th and 75th percentiles)	13 ± 10
Measured RMR (kcal), mean ± SD	2125 ± 501
Predicted RMR (kcal), mean ± SD	2210 ± 432

(BIA = Bioelectrical Impedance Analysis, MRI = Magnetic Resonance Imaging, RMR = Resting Metabolic Rate, SD = Standard Deviation).

**Table 2:** Pearson correlation coefficients between RMR and age, anthropometric data, and body composition. (* *p* sign).

Study Population Characteristics	Coef.	*p*-Value
Age (years)	−0.39	0.004 *
BMI (kg/m^2^)	0.43	0.0008 *
Fat mass (%, from BIA)	−0.06	0.69
Muscle mass (kg, from BIA)	0.78	<0.001 *
Visceral fat (kg, from BIA)	0.61	<0.001 *
Liver fat (% from MRI)	0.23	0.09
Predicted RMR (kcal)	0.78	<0.001 *

**Table 3:** Results (coefficients, 95% CI) from stepwise multiple linear regression applied to assess the effect of body composition factors on the RMR.

	Model 1 *	Model 2 **	Model 3 ***
Sex	−635.6 (−872.8, −398.4)	−582.3 (−809.6, −355.1)	−225.1 (−684.4, 234.3)
Age	-	−13.1 (−22.7, −3.4)	−5.5 (−14.2, 3.3)
Muscle mass	-	-	24.1 (9.3, 39.0)
Fat mass	-	-	7.4 (−12.2, 26.9)
Liver fat	-	-	−0.3 (−10.6, 9.9)

* Model 1: Basic model with only one predictor (sex). ** Model 2: Adds a second predictor (age). *** Model 3: Includes additional variables (body composition parameters).

## Data Availability

All data generated or analyzed during this study are included anonymously in this published article.
